# Independent Measures of Utricular Function: Ocular Vestibular Evoked Myogenic Potentials Do Not Correlate With Subjective Visual Vertical or Fundus Photographic Binocular Cyclorotation

**DOI:** 10.3389/fneur.2021.658419

**Published:** 2021-04-14

**Authors:** Sarah Hösli, Dominik Straumann

**Affiliations:** ^1^Department of Neurology, University Hospital Zurich and University of Zurich, Zurich, Switzerland; ^2^Clinical Neuroscience Center, University Hospital Zurich, Zurich, Switzerland

**Keywords:** ocular vestibular evoked myogenic potentials, subjective visual vertical, binocular cyclorotation, otolith organs, utricle, vertigo, dizziness

## Abstract

Ocular vestibular evoked myogenic potentials (oVEMPs), subjective visual vertical (SVV), and fundus photographically measured binocular cyclorotation (BCR) are diagnostic tests to assess utricular function in patients with vertigo or dizziness. In 138 patients with chronic vertigo or dizziness, we asked whether the asymmetry ratio of oVEMP (normal, right side pathological, left side pathological) could predict the SVV deviation (normal, rightward deviation, leftward deviation) or BCR (normal, cyclorotation to the right, cyclorotation to the left). There was no correlation between oVEMP and SVV and between oVEMP and BCR, while SVV and BCR correlated highly. Although both oVEMP and SVV measure aspects of utricular function, our findings demonstrate that oVEMP and SVV are not redundant and may reflect different utricular pathologies. The role of fundus photographic BCR may be relegated to only confirm unclear SVV results in vestibular diagnostic workup.

## Introduction

In patients with vertigo or dizziness, it is common to apply a comprehensive battery of auxiliary tests that help identify underlying disorders within the vestibular labyrinth, the vestibular nerve, or central vestibular networks. These vestibular tests include assessments of both semicircular canal and otolith functions (1). Frequently used otolith tests are vestibular evoked myogenic potentials (VEMPs), subjective visual vertical (SVV), and fundus photography of static binocular cyclorotation (BCR). While VEMPs are elicited by dynamic stimulation (vibration or sound) of utricular (ocular VEMPs) or saccular (cervical VEMPs) hair cells, SVV and BCR reflect the orientation of the sensed static gravito-inertial vector relative to the head in the coronal plane (2–4). Theoretically, a global hypofunction of the utricle or its afferents on one side should lead to a reduction of ocular VEMPs (oVEMPs) on the contralateral side (crossed reflex), an ipsilateral tilt of SVV, and an ipsitorsional BCR.

Test results of oVEMPs, SVV, and BCR reflect the function of different types of hair cells situated in the utricular macula. Type 1 cells are located mainly in the striola and respond to dynamic stimulation by vibration or sound. Type 2 cells are in the peripheral zones of the macula and respond to static stimulation such as constant orientation of the gravito-inertial vector relative to the head (5). While the readout of oVEMP and BCR is eye position, SVV is a psychophysical measure of the perceived earth-vertical, which subjects indicate by orienting a luminous line in otherwise complete darkness.

It was shown that SVV tilt from true earth-vertical is largest in the acute phase of a vestibular deficit, while on subsequent measurements, SVV gradually becomes close to normal again over the course of several months (6–8). This normalization of SVV is most likely due to central compensation mechanisms (9–11). The normalization of the SVV together with the unchanged oVEMP pathology may reflect the well-known pattern that high-frequency vestibular reflexes, which are transmitted along short-latency pathways with few synapses (e.g., the head-impulse vestibulo-ocular reflex), are less compensated in the chronic stage than low-frequency vestibular reflexes (e.g., caloric nystagmus) (12).

Nevertheless, asymmetries of static utricular function might still be apparent in the chronic state: When chronic patients after vestibular neuritis with normal SVV values with the head upright are roll-tilted with their heads toward the affected ear or eccentrically rotated about an earth-vertical axis passing through the unaffected ear, SVV deviations from earth-vertical are significantly larger than in healthy subjects (13–15).

In this study, we set out to analyze the association of SVV, oVEMP, and fundus photographic BCR data in a population of unselected vertigo patients who were consecutively seen in a tertiary vertigo center. We specifically asked whether there was a direct correlation between normal and side-specific pathological SVV, oVEMP, and BCR data. We also explored whether compensated SVV and BCR together with remaining oVEMP asymmetry are common patterns in chronic vertigo patients.

## Materials and Methods

### Patients

**Figure 1:**
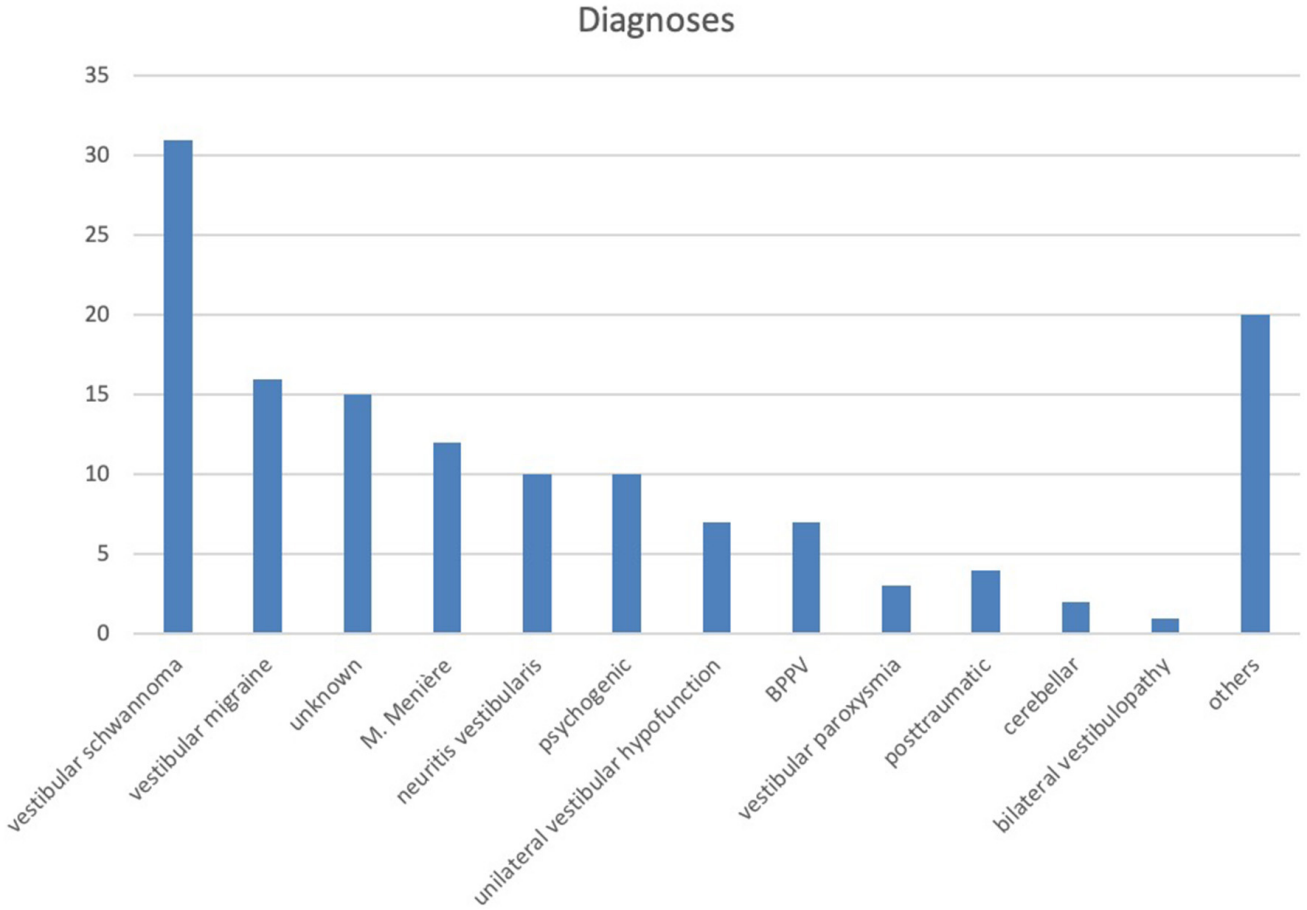
Distribution of diagnosis among patients who fulfilled the inclusion criteria. Others: polyneuropathy, ocular vertigo, mal de debarquement, orthostatic dizziness, cerebral bleeding, meningioma, Wernicke's encephalopathy.

In total, 318 patients who were seen at the Center for Vertigo and Neurological Vision Disorders, University Hospital Zurich, during the years 2017 and 2018 were screened for the study. The inclusion criteria for patients were as follows: (i) complete testing of the SVV, (ii) complete testing of oVEMP, and (iii) a signed general consent form from the hospital to use their personal data for research purposes. Among them, 178 patients were excluded because of missing general consent form and two patients were excluded because of incomplete data. Then, 138 consecutive patients (mean age: 52.8 years ± 16 SD) who fulfilled the inclusion data were selected. Here, 76 (56%) patients were male, 60 (44%) female. The diagnostic workup took place in the subacute or chronic stage after the beginning of vestibular symptoms. Final diagnosis was made after taking patient history, clinical findings, diagnostic vestibular workup, and (if obtained) MRI into consideration. The most common diagnoses were vestibular schwannomas, vestibular migraines, dizziness of unknown origin, and Menière's disease (Figure 1). If patients additionally underwent fundus photography, BCR data were also included in the analysis. The protocol was approved by the Ethics Committee Zürich (BASEC-Nr. 2017-020119).

### Subjective Visual Vertical

SVV was measured with an in-house constructed apparatus (built and programmed by U. Scheifele). For SVV measurements, patients were asked to sit on a chair in complete darkness to exclude a visual reference for upright. All data were acquired with binocular vision. A luminous arrow was projected on a circular screen (diameter: 0.4 m) in front of the patient (distance: 1.20 m). At the beginning of every trial, the luminous arrow pointed in a random direction. Patients were then asked to move the arrow into an orientation that was perceived as the perfectly earth-vertical with the arrowhead directed upward. Patients changed the orientation of the arrow by turning on the knob of a potentiometer. By pressing a button, patients confirmed the final orientation of the arrow at the end of every trial. The difference between the perceived and the true vertical was digitally recorded. Each session consisted of six trials over which the data were averaged. Based on normative data (1.2 ± 0.4 SD), an SVV deviation of more than 2.2 degrees from true vertical was considered pathological. The sign of SVV deviations to the right was defined positive.

### Ocular Vestibular Evoked Myogenic Potentials

oVEMPs were measured with a Viking V system (Nicolet Biomedical, USA) and elicited with a mini-shaker (4,810, amplifier 2,706; Bruel and Kjaer, Naerum, Denmark). Patients in supine position were visually fixing on a small object (a small model of the Earth) hanging from the ceiling (eye-to-object distance: 1.30 m). The object was positioned such that patients had to elevate the eyes by about 20 degrees relative to the straight-ahead position. The handheld mini-shaker provided bone-conducted vibration stimuli to the forehead. Here, 500-Hz stimulus vibrations with a repetition of 3.1 times per second were conducted for ~32 s. The rise/fall time was 0 ms. Muscle activity was measured with surface electrodes placed underneath each eye. Reference electrodes were placed below the active electrodes, while a grounding electrode was placed on the patient's chin. After a set of 100 repetitive stimuli, the average amplitude of the oVEMP was calculated and determined by the difference of the negative potential peak 10 ms after stimulus onset (N10) and the positive potential peak 15 ms after stimulus onset (P15). The average amplitude (A) over two sets of measurements for each eye was used to calculate the asymmetry ratio (AR) using the Jongkees formula:

(1) $$\begin{array}{r} AR=\frac{A\;right\;eye\;-A\;left\;eye}{A\;right\;eye+A\;left\;eye}\times \;100 \end{array}$$

When the response to a stimulus was absent, the size of the amplitude was defined as 0, leading to an AR of 100% to the side of the pathology. By definition, a positive value reflects a hypofunction of the right utricle. Based on normative data (15.5 ± 11% SD), AR equal to or > 30% was defined as pathological. Since all data were acquired in the subacute or chronic stage, we considered the effect of pathologies with an increase in oVEMP amplitude in the hyperacute setting [e.g., Menière's disease as described by Young et al. (16)] to have a minimal effect in our study population.

### Binocular Cyclorotation

To measure the cyclorotation (CR), a non-mydriatic retinal camera was used (Topcon TRC-NW400, Japan). Fundus photographs of each eye were taken during a period of fixating a central target while the head was placed in a perfectly upright position. Using a computer program, a straight line was drawn through the center of the papilla and the macula. By measuring the angle between this line and an earth-horizontal line, the CR of each eye was determined. The BCR of the fundus was calculated using the following formula:

(2) $$\begin{array}{r} \;BCR=\frac{CR\;right\;eye\;-\;CR\;left\;eye}{2} \end{array}$$

By definition, positive values represent a vestibular hypofunction of the right side. Based on normative data in the literature, a fundus rotation equal to or > 1.9 degrees from zero was defined as pathological (17).

### Data Analysis

The data were extracted from the clinic information system on local hospital servers. For anonymization, the personal data of subjects were coded, and any personal data, such as names or birthdates, were not included. Results of diagnostic tests were analyzed with Pearson's chi-square test and Pearson's correlation using SPSS statistics program (IBM, Armonk, USA). Matlab (MathWorks, Natick, MA, USA) was used for age-matching, bootstrapping, and calculating mean distributions between two groups.

## Results

### Ocular Vestibular Evoked Myogenic Potentials vs. Subjective Visual Vertical

Among the patients, 43 patients showed pathological SVV test results, 16 of them paired with pathological oVEMP asymmetries to either side. SVV was normal in 95 patients; 61 of these patients also had normal, i.e., symmetric, oVEMP. Considering the laterality of the pathology, there was no correlation (*p* > 0.05, Fisher's exact test; Table 1) between normal and pathological SVV and oVEMP measurements.

**Table 1:** Cross table of SVV and oVEMP.

			**oVEMP**		
			**Asymmetry to the left**	**Normal**	**Asymmetry to the right**
SVV	Tilt to the left	% of SVV (n)	9.7% (3)	64.5% (20)	25.8% (8)
	Normal	% of SVV (n)	22.1% (21)	64.2% (61)	13.7% (13)
	Tilt to the right	% of SVV (n)	33.3% (4)	58.3% (7)	8.3% (1)

*SVV, subjective visual vertical; oVEMP, ocular vestibular evoked myogenic potential. Pearson's chi-square test: not significant (p = 0.233). Fisher's exact test (33.3% of cell count <5): 5.382 (p = 0.234)*.

**Figure 2:**
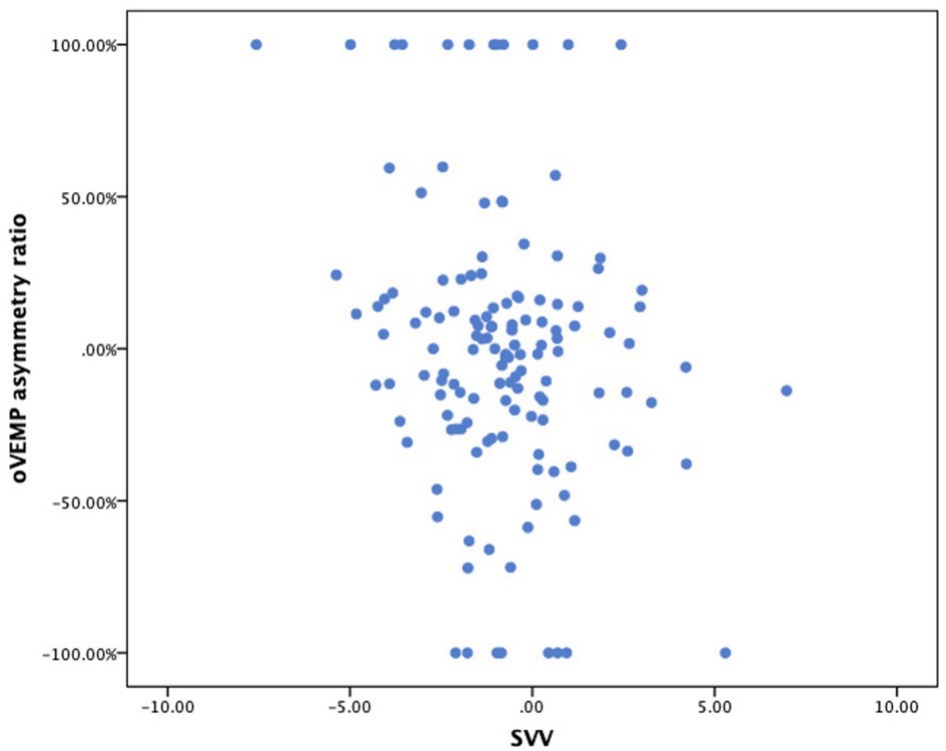
Scatterplot of SVV (x-axis) vs. oVEMP asymmetry ratio (y-axis). SVV, subjective visual vertical; oVEMP, ocular vestibular evoked myogenic potentials.

There was also no correlation between oVEMP-AR and SVV (Figure 2).

**Figure 3:**
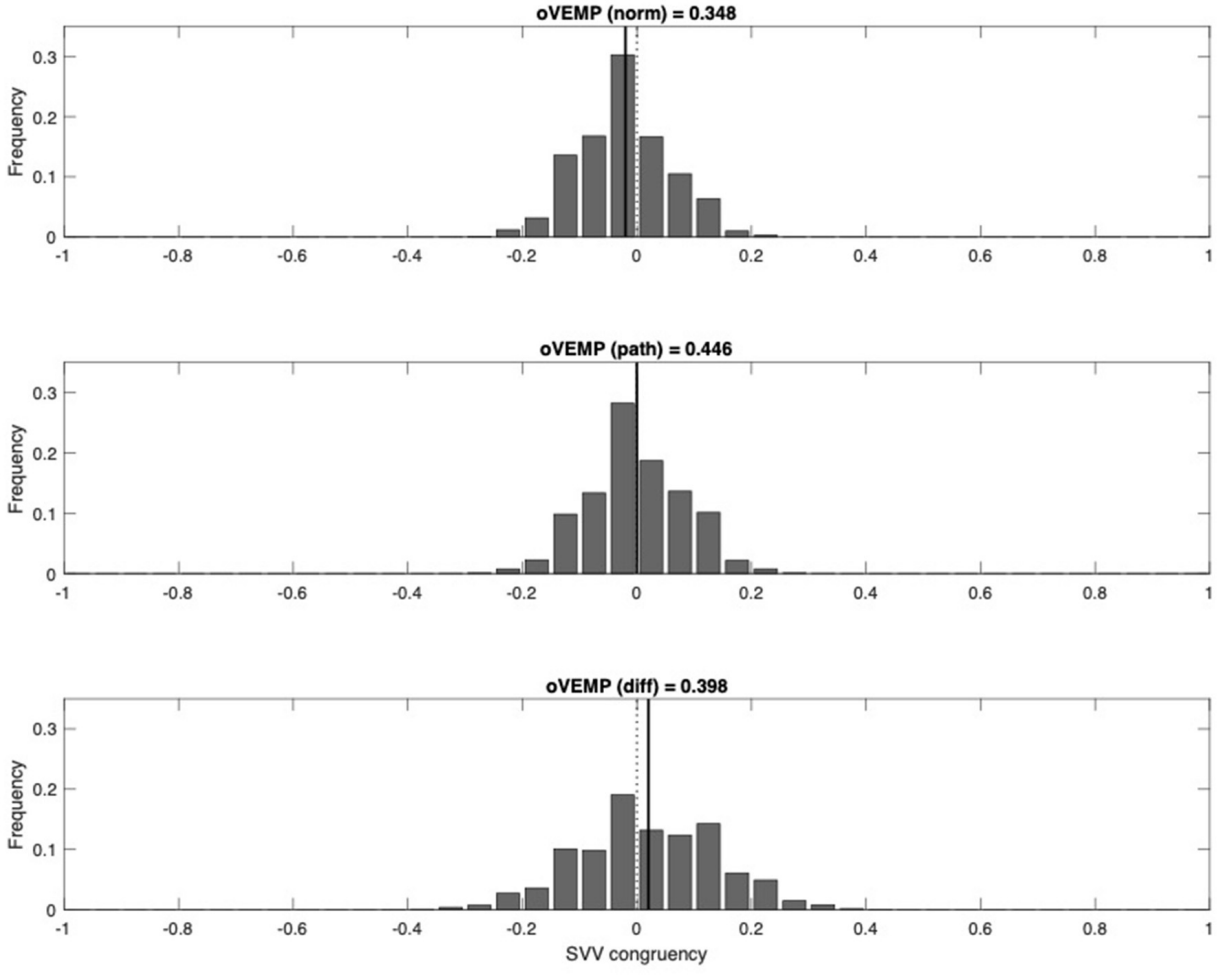
Congruency between oVEMP and SVV (upper panel, patients with normal oVEMP; middle panel, patients with asymmetric oVEMP; lower panel, difference). Groups of patients are age-matched. For each patient, a congruency value for SVV was assigned. Congruency value in patients with normal oVEMP: 0, SVV normal; 1, SVV tilted to the right; −1, SVV tilted to the left. Congruency value in patients with asymmetric oVEMP: 0, SVV normal; 1, SVV congruent with oVEMP asymmetry; −1, SVV not congruent with oVEMP asymmetry. Frequency distributions of means were obtained by bootstrapping. oVEMP, ocular vestibular evoked myogenic potential; SVV, subjective visual vertical.

In a second analysis, the hypothesis of a common chronic pattern with central compensation of SVV and BCR (low frequency), but not of oVEMP (high frequency), was explored. Among the patients, two age-matched groups were selected with an iterative algorithm for minimal age difference, one group with normal oVEMP and one group with pathological oVEMP results. In the first group *(oVEMP p norm)*, normal oVEMP would combine with pathological SVV on either the left (−1) or the right (+1) side, or with normal SVV (0). When bootstrapping the mean of this group, we would therefore expect this value to show a normal distribution around 0. The second group *(oVEMP p path)* contains pathological oVEMP values and the corresponding SVV values. Congruent pathological SVV values were assigned to the value +1, non-congruent SVV to −1, normal SVV to 0. Bootstrapping the mean of this second group, the normal distribution of SVV would be shifted in the positive direction, if pathological oVEMP were to show congruent pathological SVV values. Figure 3 shows that both groups (patients with normal oVEMP and patients with pathological oVEMP asymmetries) show a normal distribution around 0. Such pattern agrees with the hypothesis that SVV may become normal while oVEMP can still be asymmetric.

**Figure 4:**
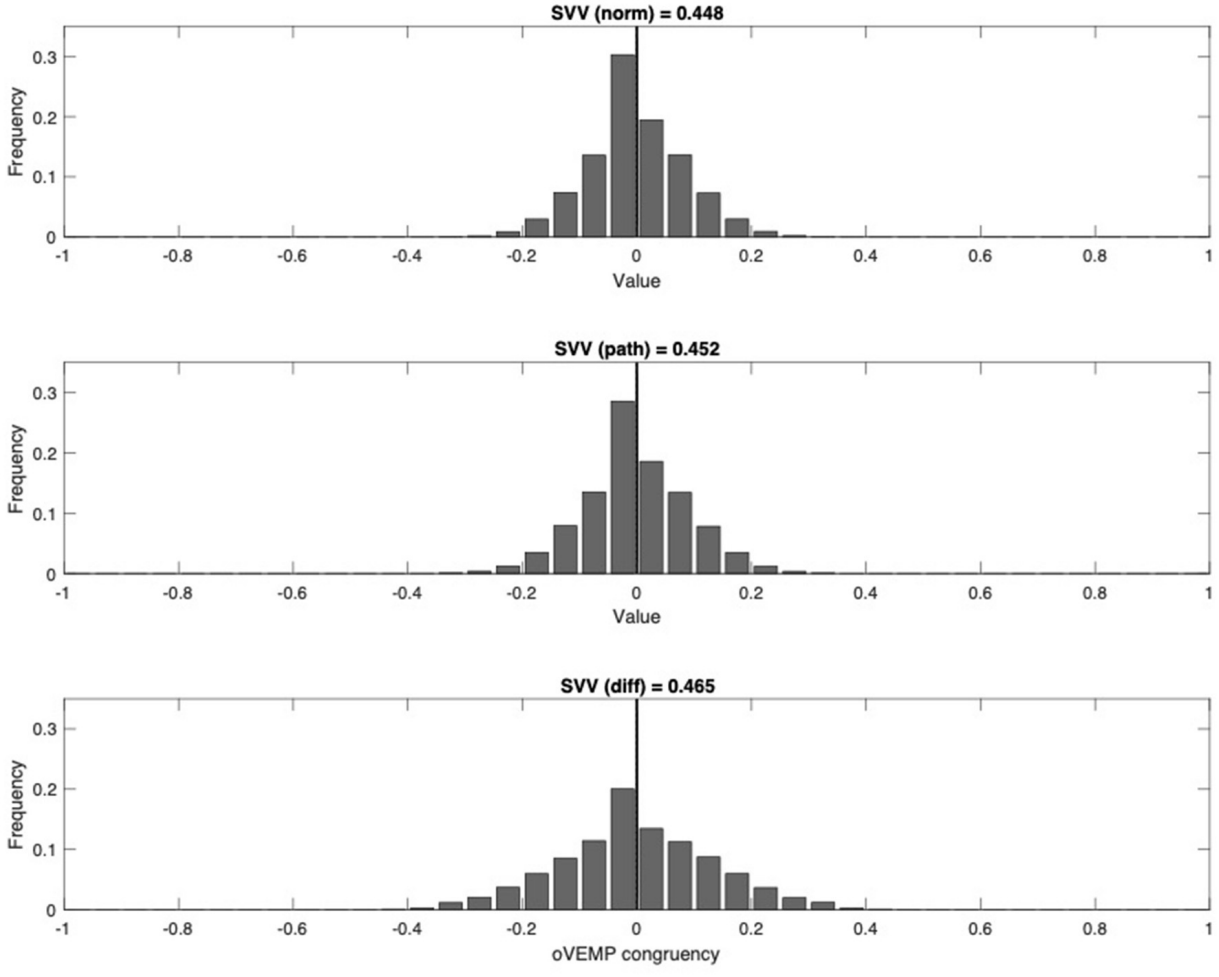
Congruency between SVV and oVEMP (upper panel, patients with normal SVV; middle panel, patients with pathologic SVV; lower panel, difference). Groups of patients are age-matched. For each patient, a congruency value for oVEMP was assigned. Congruency value in patients with normal SVV: 0, oVEMP normal; 1, oVEMP tilted to the right; −1, oVEMP tilted to the left. Congruency value in patients with pathologic SVV: 0, oVEMP normal; 1, oVEMP congruent with pathological SVV; −1, oVEMP not congruent with pathological SVV. Frequency distributions of means were obtained by bootstrapping. oVEMP, ocular vestibular evoked myogenic potential; SVV, subjective visual vertical.

The same analysis was also applied to SVV values and their corresponding oVEMP value. Again, among the patients, two age-matched groups were selected with an iterative algorithm for minimal age difference, one group with normal SVV and one group with pathological SVV results. In the first group *(SVV p norm)*, normal SVV results would combine with pathological oVEMP on either the left (−1) or the right (+1) side, or with normal oVEMP (0). When bootstrapping the mean of this group, we would therefore expect this value to show a normal distribution around 0. The second group *(SVV p path)* contains pathological SVV values and the corresponding oVEMP values. Congruent pathological SVV values were assigned to +1, non-congruent SVV values to −1, and normal SVV values to 0. We expected that pathological SVV would generally combine with a congruent oVEMP asymmetry, i.e., a shift in the normal distribution of *SVV p path* toward 1 when compared to *SVV p norm*. Figure 4 shows that this shift cannot be observed; therefore, the expected congruency between pathological SVV with pathological oVEMP could not be demonstrated in our data (Figure 4).

### Binocular Cyclorotation vs. Subjective Visual Vertical

Here, 99 out of the 138 patients had also undergone fundus photography to measure BCR. Since both SVV and BCR result from a static, i.e., low-frequency, asymmetry of the utricular signals, we expected to find a direct correlation (18). Among them, 36 patients showed a pathological SVV, 19 of them also showing abnormal results in the fundus rotation. However, 63 patients had normal SVV results, 49 of them also showing normal BCR. The correlation between the SVV results and the fundus rotation was highly significant (*p* <0.001, Fisher's exact test; Table 2).

**Table 2:** Cross table of SVV and BCR.

			**BCR**		
			**Tilt to the left**	**Normal**	**Tilt to the right**
SVV	Tilt to the left	% of SVV (n)	51.9% (14)	44.4% (12)	3.7% (1)
	Normal	% of SVV (n)	17.5% (11)	77.8% (49)	4.8% (3)
	Tilt to the right	% of SVV (n)	0.0% (0)	55.6% (5)	44.4% (4)

*SVV, subjective visual vertical; BCR, binocular cyclorotation. Pearson's chi-square test: significant (p = 0.000). Fisher's exact test (33.3% of cell count <5): 22.419, p < 0.001. Cramer V = 0.395*.

**Figure 5:**
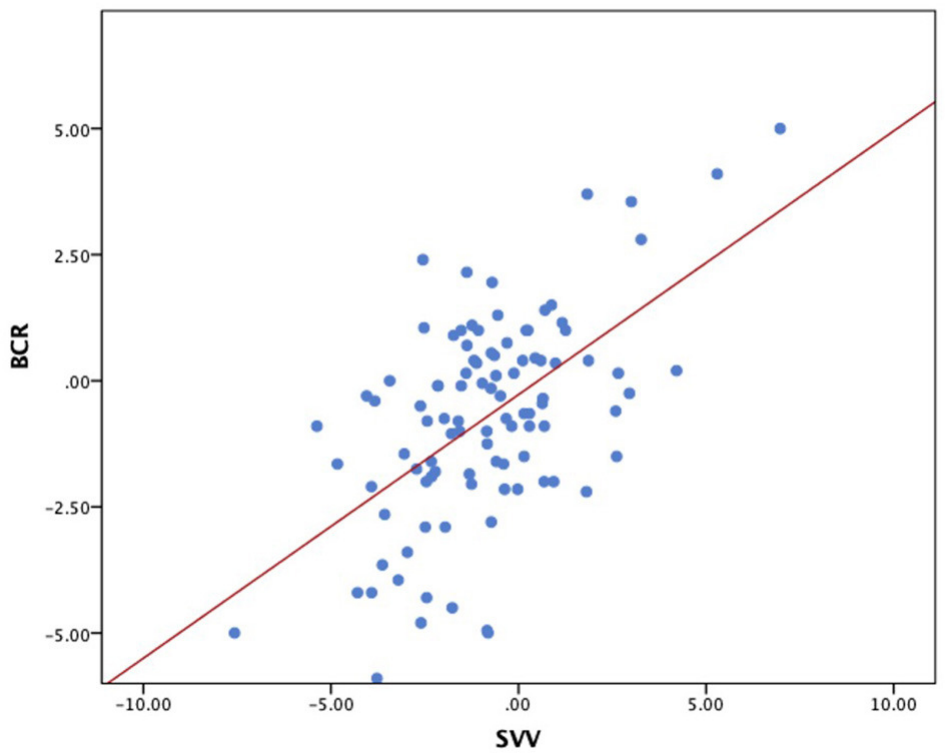
Scatterplot of SVV (x-axis) vs. BCR (y-axis). Regression line: BCR = 0.27 + 0.52 × SVV. Spearman correlation: R^2^ = 0.326; *p* < 0.001. SVV, subjective visual vertical; BCR, binocular cyclorotation.

Figure 5 depicts the scatterplot of the significant correlation between SVV and BCR.

### Ocular Vestibular Evoked Myogenic Potentials vs. Binocular Cyclorotation

**Figure 6:**
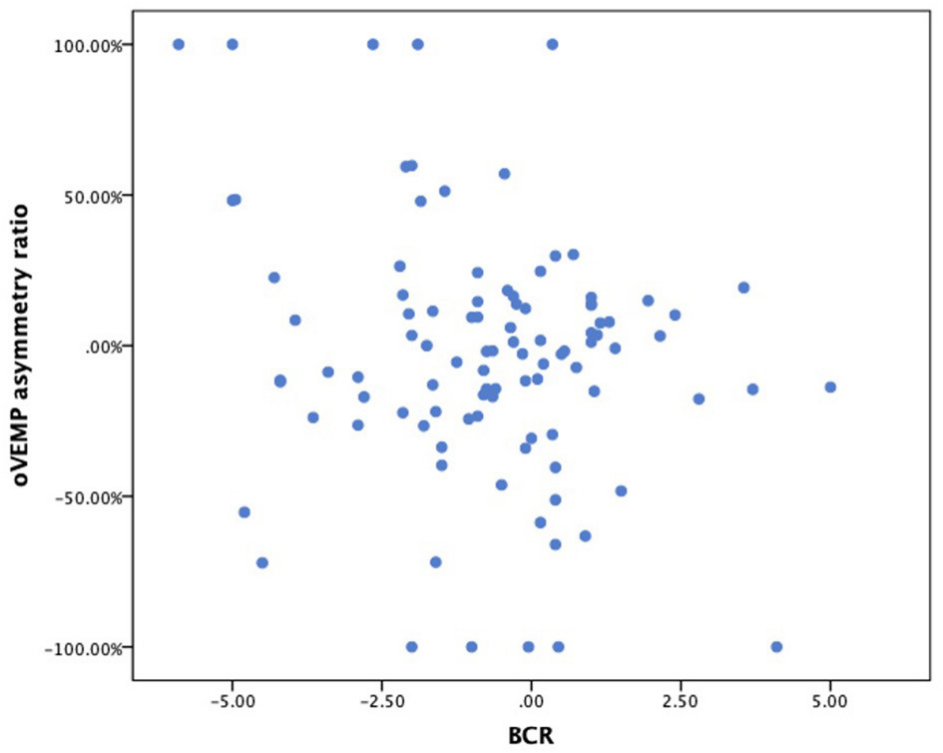
Scatterplot of BCR (x-axis) vs. oVEMP asymmetry ratio (y-axis). BCR, binocular cyclorotation; oVEMP, ocular vestibular evoked myogenic potential.

After finding a direct correlation between SVV and BCR, patients' oVEMP data were also compared with the BCR as a control for the initial SVV and oVEMP comparison. Here, 33 patients were measured with abnormal oVEMP, 12 of them also showing pathological fundus rotation. Of the 66 patients with normal oVEMP, 46 also measured normal fundus rotation. The direct correlation between oVEMP and BCR (taking into account the laterality of the pathology) was significant (*p* < 0.05, Fisher's exact test; Table 3), but with a low Cramer's index. There was no significant correlation between oVEMP and BCR, as shown in Figure 6 (Spearman-rho correlation coefficient −0.114, *p* = 0.261).

**Table 3:** Cross table of oVEMP and BCR.

			**BCR**		
			**Tilt to the left**	**Normal**	**Tilt to the right**
oVEMP	Asymmetry to the left	% of oVEMP (n)	15.8% (3)	78.9% (15)	5.3% (1)
	Normal	% of oVEMP (n)	20.9% (14)	68.7% (46)	10.4% (7)
	Asymmetry to the right	% of oVEMP (n)	61.5% (8)	38.5% (5)	0.0% (0)

*oVEMP, ocular vestibular evoked myogenic potential; BCR, binocular cyclorotation. Pearson's chi-square test: significant (p = 0.022). Fisher's exact test (44.4% of cells expected count < 5): 9.239, p = 0.038. Cramer V = 0.243*.

### Diagnosis-Specific Measurements

**Figure 7:**
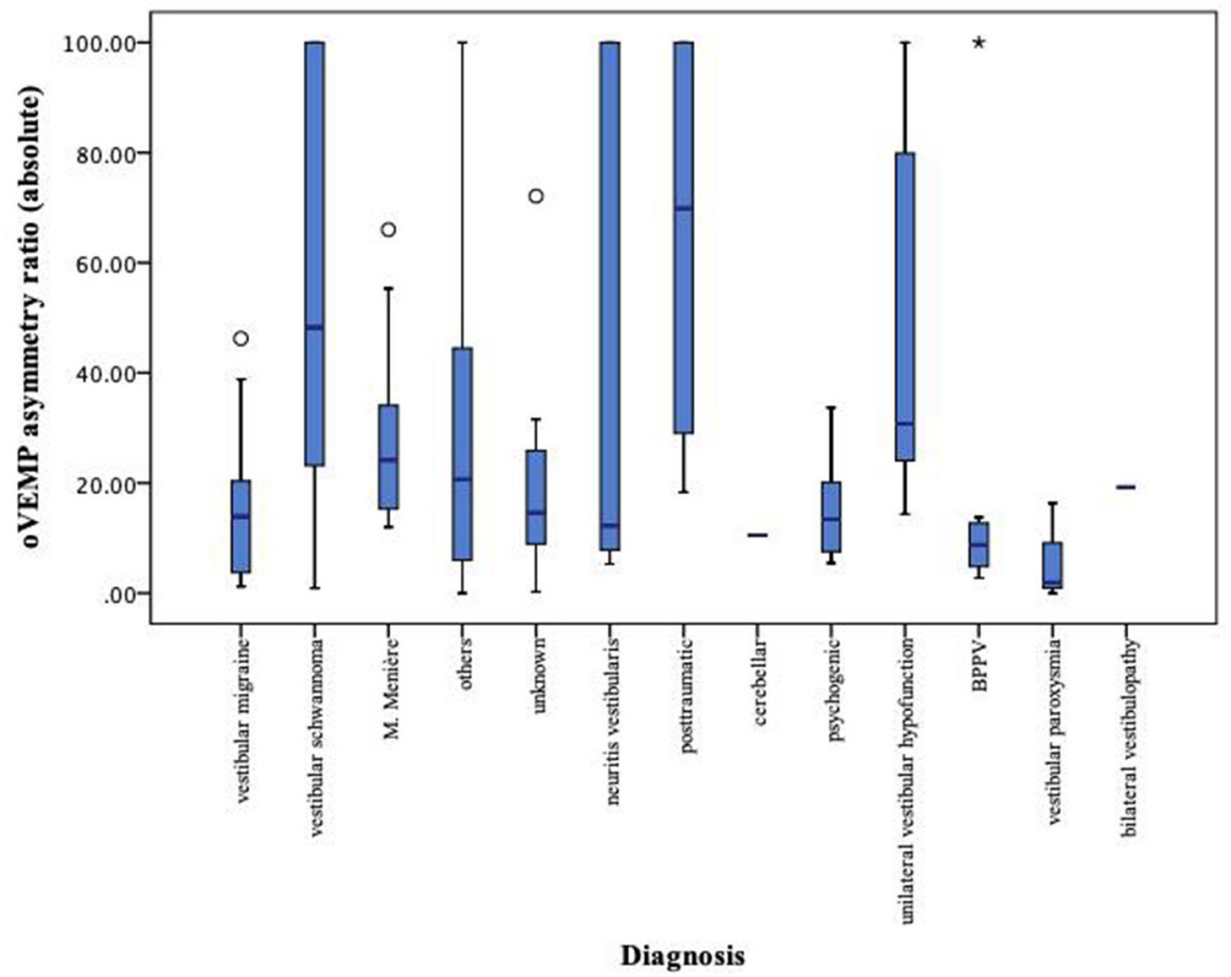
Boxplot of oVEMP measurements for each diagnosis separately. oVEMP, ocular vestibular evoked myogenic potential; left-sided values (original data = <0) and right-sided values (original data = >0) both expressed as positive values. *Extreme value, below quartile 1 or above quartile 3.

**Figure 8:**
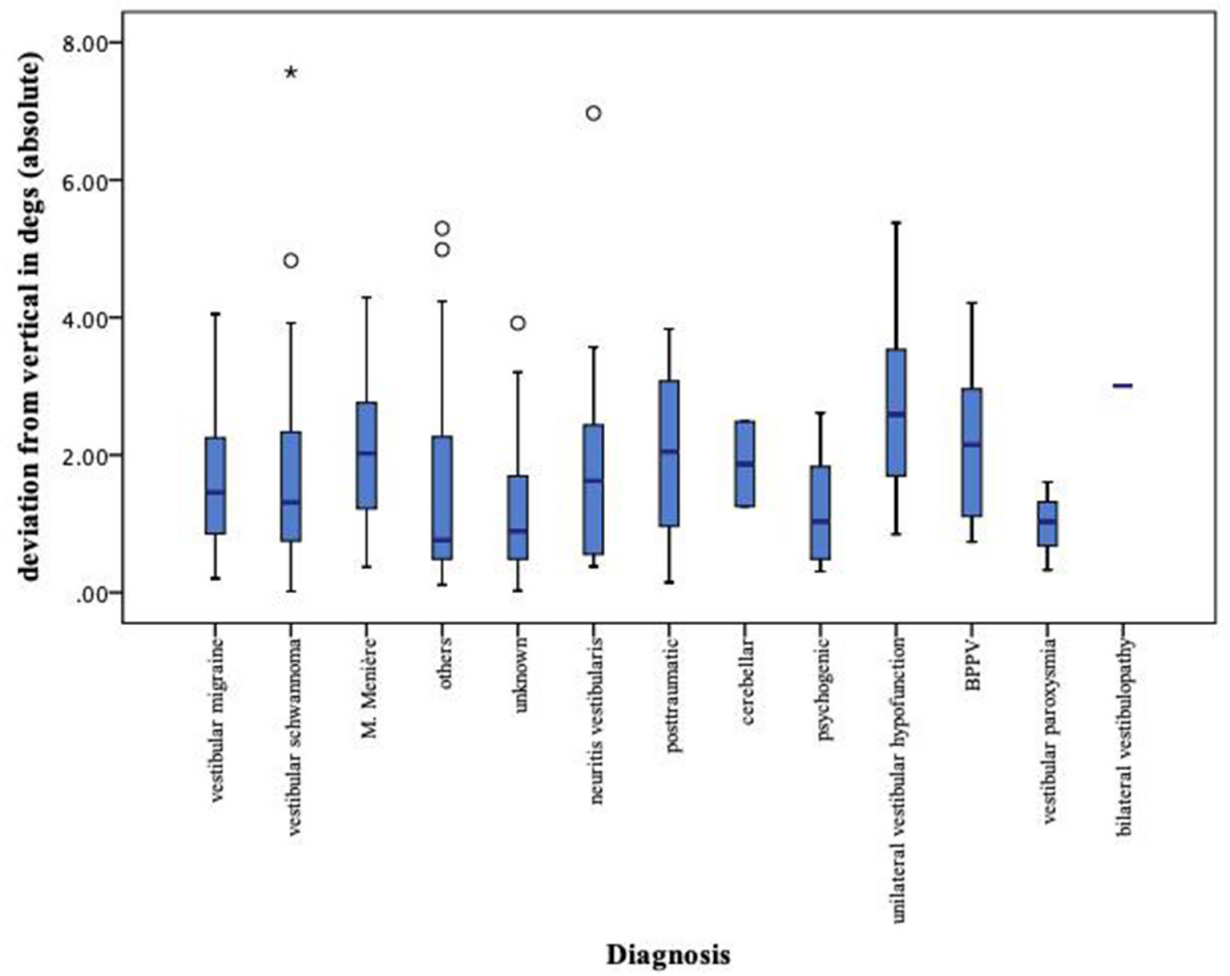
Boxplot of SVV measurements for each diagnosis separately. SVV, subjective visual vertical; left-sided values (original data = <0) and right-sided values (original data = >0) both expressed as positive values. *Extreme value, below quartile 1 or above quartile 3.

The data were analyzed by each diagnosis separately. Here, it became apparent that for oVEMP and SVV data, there is no significant intervariability between each group and that the range of pathological measurements is large (Figures 7, 8).

## Discussion

This study shows that in an unselected sample of patients with vertigo or dizziness, oVEMP results (normal, right side pathological, left side pathological) were not predictive of SVV results (normal, deviated to the right, deviated to the left) or BCR results (normal, CR to the right, CR to the left). There were no correlations between oVEMP and SVV and between oVEMP and BCR. The possible chronic pattern of persistent oVEMP asymmetry and normalized (i.e., compensated) SVV, however, is compatible with our findings. Moreover, SVV and BCR results correlated highly, as it has previously been shown by Curthoys et al. (18) and Schmidt et al. (19).

Putting the study into context with previous work, Nagai et al. (20) had consistent results, not finding SVV and oVEMP results to correlate in patients with different acute pathologies of the inner ear. Ogawa et al. (21) also did not find a significant correlation in the rates of abnormal SVV and abnormal oVEMP results when examining patients over the course of 20 days after the onset of vestibular neuritis. In contrast, the study by Lin and Young (22) did find a significant correlation between SVV and oVEMP in healthy subjects as well as in patients with Menière's disease. In a 2016 conducted study, Taylor et al. (23) found concordant results between subjective visual horizontal (an alternative static test to the SVV) and oVEMP in the acute setting of vestibular neuritis in 38 patients. In the follow-up of 16 of these patients 12 month later, a similar recovery pattern was reported for the two tests (23). Because of compensation mechanisms that are more effective in static vestibular function tests, Magliulo et al. (24) postulated that oVEMP can be of better use as a prognostic parameter. A lack of correlation for oVEMP and BCR has also been described in healthy controls and in patients with vestibular neuritis by Cherchi (25) and Zalewski et al. (26).

It can be hypothesized that the incongruent SVV and oVEMP results are explained by the different functioning of the two cell types found in otolith organs, one effective for static stimulation, the other for dynamic stimulation (5, 27). This differentiation between the computation of static and dynamic vestibular signals not only applies to a peripheral level but also is of importance in central vestibular structures (28, 29). As the segregation of static and dynamic vestibular signals carries over to central structures, separate compensation mechanisms could be plausible. Further, it can be hypothesized that pathologies of the utricle could affect the two cell types differently and therefore lead to divergent results when testing the static and dynamic systems. This is supported in the findings that, in humans, the cells of the macula show different involvement after being exposed to external influences. In the study of Lyford-Pike et al. (30), it has been demonstrated that type 1 cells show greater gentamicin accumulation than type 2 cells when exposed to the antibiotic.

In our study, we examined the correlation between oVEMP and SVV in a relatively large number of patients with different diagnoses, which so far has not been reported by others. In contrast to previous studies, the laterality of SVV and oVEMP asymmetries was also taken into account, an important factor when looking for a correlation between the two tests. Moreover, the highly significant correlation between SVV and BCR results provides good evidence that BCR data can usually be used to confirm the SVV results, if they are in doubt. This of course is limited to vestibular diagnostic workup, since BCR remains an important tool in the evaluation of neuro-ophthalmological conditions (31).

The main limitation of the study lies in the singular measurement per patient. To assess the hypothesis of SVV results changing from initial pathological findings to a state of compensation, a prospective study with set examination points after a first measurement during the acute stage would be interesting, as it would give insight to changes from the acute to the chronic stages.

The findings from this study support the use of multiple vestibular tests in patients with vertigo or dizziness. We demonstrated that oVEMPs are an important expansion of the diagnostic workup for utricle function and cannot replace SVV testing. Since there is a strong correlation between SVV and BCR, BCR measurements might be of use in cases of unclear SVV results.

## Data Availability Statement

The datasets presented in this article are not readily available because this would jeopardize patient confidentiality. Requests to access these datasets should be directed to sarah.hoesli@uzh.ch.

## Ethics Statement

The studies involving human participants were reviewed and approved by Ethics committee Zurich, Switzerland. The patients/participants had provided a written general consent for the use of their data for research purpose.

## Author Contributions

DS designed and developed the concept of the study. SH gathered and organized the data and wrote the manuscript in consultation with DS. SH and DS contributed to the analysis of the data and interpretation of the results.

## Conflict of Interest

The authors declare that the research was conducted in the absence of any commercial or financial relationships that could be construed as a potential conflict of interest.
